# Comparison of patient- and clinician-reported outcome measures in lower back rehabilitation: introducing a new integrated performance measure (t2D)

**DOI:** 10.1007/s11136-021-02905-2

**Published:** 2021-06-15

**Authors:** Andrej Zdravkovic, Vincent Grote, Michael Pirchl, Martin Stockinger, Richard Crevenna, Michael J. Fischer

**Affiliations:** 1 Department of Physical Medicine, Rehabilitation and Occupational Medicine, Medical University of Vienna, Vienna, Austria; 2 Ludwig Boltzmann Institute for Rehabilitation Research, Reizenpfenninggasse 1, 1140 Vienna, Austria; 3 Vamed Rehabilitation Center Kitzbühel, Hornweg 32, 6370 Kitzbühel, Austria; 4 Hannover Medical School MHH, Clinic for Rehabilitation Medicine, Hannover, Germany; 5 Otto Loewi Research Center, Division of Physiology, Medical University of Graz, Graz, Austria

**Keywords:** Patient-reported outcome measures, Clinician-reported outcome measures, Performance score, Orthopedic rehabilitation, Inpatient, Lower back pain

## Abstract

**Purpose:**

Patient- and clinician-reported outcome measures (PROMs, CROMs) are used in rehabilitation to evaluate and track the patient’s health status and recovery. However, controversy still exists regarding their relevance and validity when assessing a change in health status.

**Methods:**

We retrospectively analyzed the changes in a CROM (Fingertip-To-Floor Test – FTF) and PROMs (ODI, HAQ-DI, NPRS, EQ5D) and the associations between these outcomes in 395 patients with lower back pain (57.2 ± 11.8 years, 49.1% female). We introduced a new way to measure and classify outcome performance using a distribution-based approach (t2D). Outcome measures were assessed at baseline and after 21 days of inpatient rehabilitation.

**Results:**

Overall, the rehabilitation (Cohens *d* = 0.94) resulted in a large effect size outcome. Medium effect sizes were observed for FTF (*d* = 0.70) and PROMs (*d* > 0.50). Best performance rating was observed for pain (NPRS). We found that 13.9% of patients exhibited a deterioration in the PROMs, but only 2.3%, in the FTF. The correlation between the PROMs and FTF were low to moderate, with the highest identified for HAQ-DI (rho = 0.30*–*0.36); no significant correlations could be shown for changes. High consistency levels were observed among the performance scores (t2D) in 68.9% of the patients.

**Conclusions:**

Different and complementary assessment modalities of PROMs and CROMs can be used as valuable tools in the clinical setting. Results from both types of measurements and individual performance assessments in patients provide a valid basis for the meaningful interpretation of the patients’ health outcomes.

*Trial registration*. This clinical study was entered retrospectively on August 14, 2020 into the German Clinical Trials Register (DRKS, registration number: DRKS00022854).

## Introduction

Low back pain (LBP) is highly prevalent in the general population [1] and has been ranked sixth globally in terms of overall disease burden [2]. Exercise therapy, which is often prescribed for LBP, has been found to positively affect pain levels, as well as physical functioning in LBP patients [3]. However, it is still unclear whether subjective, patient-reported outcome measures (PROMs) reflect the actual course of convalescence in LBP rehabilitation, if this is better achieved by applying objective, clinician-reported outcome measures (CROMs) alone, or if a combination of PROMs and CROMs should be applied. The responsiveness and validity of different PROMs have been studied in LBP patients, although usually not in relation to changes in CROMs [4–6]. Although several PROMs may exhibit certain methodological limitations [7], they represent a necessary tool that can be used to involve patients in therapy [6] and help predict the socioeconomic cost of LBP [8].

Despite some controversy regarding the optimal methods and techniques for the measurement of lumbar function, lumbar flexion tests form the cornerstone of assessments in impairment due to LBP [9]. Apart from the historical reasons for their use, measurements of spinal flexion have been shown to correlate highly with the degree of disability [10]. The Fingertip-to-Floor test (FTF) and the Schoeber test represent commonly used CROMs which have been validated for the LBP patient population [11, 12].

In this and in other patient populations, however, researchers have observed a divergence between self-reported outcome measurements and performance outcomes. They have also identified multiple factors that can influence this discrepancy in outcomes, including gender, education and mood [13–16]. This discrepancy needs to be considered in light of the different health conditions of the patients, in order to find a performance evaluation that also adequately considers those patients who already show satisfactory results (ceiling effects). These findings indicate that further research is warranted to ascertain the relative changes in PROMs and CROMs, as well as their mutual coherence. Furthermore, the simplicity of reporting outcomes in the clinical setting should not be neglected, and user-friendly options should be developed, in order to facilitate the implementation of research findings into clinical practice. To this end, in this paper, we describe a new way to evaluate outcomes and the results of an analysis of the PROMs and CROMs of LBP patients before and after a course of multidisciplinary inpatient rehabilitation at a center in Tyrol, Austria.

## Methods

### Study aim, design and setting

LBP patients were treated at a specialized orthopedic rehabilitation center in Austria. The most frequent International Classification of Diseases, 10th Revision (ICD-10), diagnoses noted at the study center were M51.1, M53.9, M54.9, M51.2 and M54.4 (all *n* > 30). The PROMs consisted of the following instruments: Oswestry Disability Index (ODI), Numeric Pain Rating Scale (NPRS), Health Assessment Questionnaire Disability Index (HAQ-DI) and the Five-Level EuroQol-5D (EQ5D-5L). The CROM used was the Fingertip-to-Floor test (FTF). In a retrospective cohort study, the changes and correlations between the CROM and PROMs were analyzed at the beginning (*t*1) and end (*t*2) of orthopedic rehabilitation. We empirically tested and present a new method that can be used to measure and stratify outcome performance. This method uses a distribution-based approach and is based on two measurements at the beginning and end of rehabilitation, the “performance score (t2D)”. The patients were fully informed about the study content and purpose and gave their written consent to participate.

### Intervention

The inpatient program lasted 21 days, as defined in the service portfolio of the Austrian social security institutions [17]. The medical treatments last on average 2–3 h per day, including exercise therapy, electrotherapy, lymphatic drainage and massage as well as hydrotherapy. These treatments amounted to at least 1800 therapy minutes during the three-week rehabilitation program. The amount of individual therapy depends on the medical history if the rehabilitation program is classified as a follow-up treatment procedure after surgery.

### Ethics approval

The Ethics Committee of the Medical University of Innsbruck approved the study protocol on August 23, 2019 (Ref: EC Nr: 1158/2019) in accordance with the current version of the Declaration of Helsinki.

### Clinician-reported outcome measure

The CROM used in the assessments was the FTF. While different techniques can be used to measure lumbar flexion, including the FTF and the Schoeber Test [12], the method used should be safe, user-friendly and time-efficient in clinical practice. We preferred the FTF, as it fulfills the mentioned criteria. The FTF tests combine spinal and hip flexion and correlate highly with radiographic measurements of lumbar flexion [11].

### Patient-reported outcome measures

PROMs are standardized, validated questionnaires that are completed by patients in order to measure perceptions of their functional status and wellbeing [18]. Outcomes reflect the overall care for a patient’s medical condition, in which professionals in multiple specialties are usually involved [19]. Professionals using PROMs as clinical tools need to be sensitive to the situation of the individual patients. PROMs can provide insights that support direct clinical decision-making and enhance experiences of care [20].

#### Numeric pain rating scale (NPRS)

Pain intensity and impairments in physical functions are associated in patients with chronic pain, and improvement in pain has been associated with improvements in functioning [21]. There are two aspects of pain, which can be evaluated independently. Firstly, the intensity or how strong the pain feels and, secondly, the affective dimension of pain or how unpleasant the pain feels [22]. Self-report measures provide the ‘gold standard’ in assessing pain, as they reflect the subjective nature of pain. The commonly used methods of rating pain include the visual analogue scale, verbal rating scales and – the method used in our study – numerical rating scales [21].

#### ODI 2.1a

The Oswestry Disability Index is an instrument used to quantify disability in patients with low back pain, which was originally described in 1980 [23]. The questionnaire has since been revised, with the current version being 2.1a [24]. It encompasses ten dimensions of disability involving pain, personal care, lifting, walking, sitting, standing, sleeping, sex life, social life and travelling [25]. The score is rated on a percentage scale, with 0% representing no disability and 100% representing the highest degree of disability. The German version used in the current study has been validated in a German-speaking population [26].

#### HAQ-DI

The Health Assessment Questionnaire was first proposed in 1980 as a comprehensive measure of a patient’s health status and patient-centered care in rheumatoid arthritis [27]. The disability dimension of the original questionnaire is widely used, as it addresses common activities of daily living. These are scored on a scale of 0–3, corresponding to “do without difficulty” and “unable to do”, respectively [27–29].

#### EQ5D-5L

The EuroQol-5D is a generic instrument used for assessing health-related quality of life. It was designed as a self-complete questionnaire. The EuroQol-5D was originally introduced with three levels of severity in 1990 by the EuroQol Group [30–32] and was subsequently expanded to include five levels to improve its responsiveness and reduce the ceiling effect [33]. The EQ5D-5L is applied to measure five dimensions of health status [34]: mobility, self-care, usual activities, pain/discomfort and anxiety/depression. In addition, the subjective overall health status is estimated using a visual analogue scale (EQ-VAS [0–100]). The five dimensions are rated on five severity levels, with 1 corresponding to “no problem” and 5 to “unable to do / extreme problems”. The value set of the EQ5D-5L (EQ5D TTO [-0.66–1.00]) for a German population has been published elsewhere [35].

### Performance score (*t*2D: *t*2 + Δ)

Taking objective measurements of physical mobility can lead to ceiling effects. For example, if a patient has good lumbar flexion (FTF test; spinal and hip flexion) at the beginning of rehabilitation, no strong increase (change) is expected during the course of the rehabilitation. In this case, although the patient’s overall performance may be good, only a slight or no increase can be measured statistically. Thus, to assess the performance of patients using each score, a new method was introduced to account for the fact that the change in scores depends on the patient’s initial functional status. The simple formula *t*2 + (*t*2 − *t*1) best reflects the performance and considers the functional status at the end of rehabilitation and improvements (changes from *t*1 to *t*2; Δ). It was possible to interpret “performance scores” using a distribution-based approach, in which the *t*2 + Δ were transformed into standardized scores for *t*1 and *t*2 with *z*-transformation.

### Statistical analysis

SPSS for Windows (version 27) was used for data analysis. For each outcome measure, score differences (Δ, changes) between the beginning (*t*1, pre-test score) and the end (*t*2, post-test score) of rehabilitation were calculated and tested for significant changes using *t*-tests. For multiple comparisons, 2 × 2 MANOVA for repeated measurements was used. *Z*-values and effect sizes for within-subjects designs were calculated (Cohen’s *d* and partial Eta-squared, η_p_^2^). Effect sizes were interpreted according to Cohen [36], while correlations between CROM and PROMs were determined using Spearman’s rank correlation coefficients (rho), Pearson product-moment correlations (*r*) or linear regression models for *t*1, *t*2, changes (Δ) and *performance scores (t*2 + Δ).

The difference between the standardized performance (*z*-)scores of PROMs and CROMs was calculated to show the level of consistency. Performance scores (*t*2D; *t*2_z_ + Δ_z_) for each outcome measure were classified as high consistent (within one/same tertile; cut-off: normalized *z*-difference between absolute value of *t2*_*z*_ + Δ_z_ of CROM and PROMs < 0.43), moderate consistent (if the scores ranged between one and two tertiles; *z*-difference within 0.43–0.97), or as low consistent and discrepant (more than two tertiles of difference between performance scores; *z*-difference > 0.97). By chance, this would result in an equivalence of 33.3% in each category, if no correlation existed between the different measured performance outcomes.

## Results

A total of 395 LBP patients who underwent a standardized rehabilitation program between January and December 2018 were included in this study. All LBP patients suffered from afflictions of the lower back and either had or had not experienced recent surgical treatment. The average age of the patients was 57.2 years with a standard deviation of 11.8 years. Among these patients, 49.1% were women. Post-intervention, statistically significant changes in the EQ5D, NPRS, HAQ, ODI and FTF were detected (all *p* < 0.001) with medium effect sizes (Cohen’s *d* = 0.55–0.70). The changes in PROMs and the FTF are shown in Table 1.

**Table 1:** Patient’s health status and changes in PROMs and the FTF

Quality-of-outcome measures		*t*1	*t*2	Δ	*p*	Cohen’s *d*_z_
CROM	FTF	**17.9 ± 15.1**	**13.2 ± 12.4**	**−4.71 ± 6.74**	***	**0.70**
	FTF [z]	**0.17 ± 1.08**	**−0.17 ± 0.88**	**−0.34 ± 0.48**	***	**0.70**
PROMs	EQ5D Health (EQ-VAS)	61.1 ± 19.3	66.7 ± 22.7	5.63 ± 22.57	***	0.25
	EQ5D TTO	0.81 ± 0.17	0.87 ± 0.15	0.06 ± 0.14	***	0.39
	NPRS	4.58 ± 2.07	3.50 ± 1.98	**−**1.09 ± 1.88	***	0.58
	HAQ	0.27 ± 0.29	0.23 ± 0.31	**−**0.04 ± 0.17	***	0.25
	ODI	22.6 ± 14.2	17.7 ± 14.2	**−**4.84 ± 8.86	***	0.55
	Mean PROMs [z]	**0.21 ± 0.97**	**−0.21 ± 0.99**	**−0.41 ± 0.63**	***	**0.66**
Overall MQO	Medical Outcome [z]	**0.23 ± 1.02**	**−0.23 ± 0.93**	**−0.45 ± 0.48**	***	**0.94**

CROM-FTF, mean of PROMs and the overall medical outcome (MQO; mean of PROMs and CROM-FTF) are highlighted in bold

Quality-of-outcome measures were documented in the discharge report at the beginning (*t*1) and at the end (*t*2) of the 21-day inpatient rehabilitation program. The PROMs consisted of Oswestry Disability Index (ODI), Numeric Pain Rating Scale (NPRS), the Health Assessment Questionnaire Disability Index (HAQ-DI) and the Five-Level EuroQol-5D (EQ5D-5L). The CROM was the Fingertip-to-Floor test (FTF), where the optimal value in this study has been defined as 0 [cm]. Differences between those measurements (difference: *t*2-*t*1) and effect sizes (Cohen’s *d*_z_) were used to evaluate recovery in rehabilitation. The level of statistical significance was reached for all outcome measures (all *p* < 0.001***; η_p_^2^_multivariate_ = 0.490)

*n* (m/f): 395 (201/194)

The percentage of patients who showed an improvement, deterioration, or no change of the outcome measures is shown in Table 2. The overall Medical Quality Outcome (MQO; mean of PROMs and CROM-FTF) revealed improvements in 68.9% of patients immediately at the end of the rehabilitation (cut-off: *z*-difference *(t*2 *– t*1*)* < -0.20; [16]). The status of 24.8% of the patients remained unchanged (0.00 ± 0.20), and the conditions of 6.3% worsened (> 0.20) between the beginning and the end of the inpatient rehabilitation. The overall improvement in MQO was around 13.12 ± 14.24 percentile points or a SMD of 0.45 ± 0.48 (*r*_t1, t2_ = 0.89; Cohen’s *d* = 0.94, 95% CI [0.83, 1.06]). The majority of the measures improved following the intervention; however, 13.7–22.5% of patients exhibited a deterioration in the individual PROMs, whereas only 2.3% experienced a deterioration in the FTF.

**Table 2:** Improvements of outcome measurements

SMD (*z*-differences) classified	*N* [%]	Better ( +) (%)	Equal ( =) (%)	Worse (−) (%)
CROM (1)	FTF^+^	**50.9**	**46.8**	**2.3**
PROMs (6)	EQ5D Health (EQ-VAS)	54.2	23.3	22.5
	EQ5D TTO	52.9	33.2	13.9
	NPRS	61.8	20.8	17.5
	HAQ^++^	31.6	54.7	13.7
	ODI	57.2	28.1	14.7
	Mean PROMs	**62.3**	**23.8**	**13.9**
Overall MQO (PROMs and CROM-FTF)		**68.9**	**24.8**	**6.3**

CROM-FTF, mean of PROMs and the overall medical outcome (MQO; mean of PROMs and CROM-FTF) are highlighted in bold

Changes between admission and discharge (categorical presentation: better, equal, worse); The threshold used was an average *z*-difference (SMD) of > 0.20. + …Results for subsample without 73 LBP Patients who had optimal FTF values (0) for *t*1 and *t*2: 62.4% better, 34.8% equal and 2.8% worse (*n*_1_ = 322); +  + … Results for the subsample also without (not the same) 73 LBP patients who had optimal HAQ scores (0) for *t*1 and *t*2: 38.8% better, 44.4% equal and 16.8% worse (*n*_2_ = 322)

Normalized changes between admission (*t*1) to discharge (*t*2) are revealed by examining the effect sizes (*z*, SMD) and the number of patients (*n* [%]), which could be improved in clinically relevant ways [16]. Based on the value distributions, the individual outcome parameters were transformed into *z*-values. By means of *z*-standardization, differently scaled quantities were summarized, and the changes were uniformly quantified. *Z*-differences from 0.00 ± 0.20 were classified as equal (no changes) [16, 36]

The correlations among the different outcome measures are shown in Table 3a–d. Although multiple measures reached the level of statistical significance (*p* < 0.001; η_p_^2^_multivariate_ = 0.490), the correlation between the FTF and the PROMs was weak (*rho* = 0.16–0.36). The strongest relation to CROM was observed for HAQ-DI (rho = 0.30*–*0.36), which was confirmed by linear regression models. The relationships at the beginning and end of rehabilitation are similar, but no correlations between changes (differences from *t*1 to *t*2) or between FTF and PROMs could be found (all rho < 0.05). In contrast, the performance scores in the PROMs showed significant correlations with the CROM-FTF performance score (*R*^*2*^_linear regression_ = 0.09; *p* < 0.001).

**Table 3:** **abcd** Intercorrelation of the measurements for *t*1, *t*2, differences (Δ) and *t*2 + Δ

(**a**) Spearman correlations (rho) for *t*1; *n* = 395						
*t*1	FTF-CROM	EQ-VAS	EQ5D TTO	NPRS	HAQ	ODI
FTF-CROM	–					
EQ5D health (EQ-VAS)	**−**0.16**	–				
EQ5D TTO	**−**0.19**	0.39**	–			
NPRS	0.18**	**−**0.36**	−0.58**	–		
HAQ	0.30**	**−**0.37**	−0.66**	0.44**	–	
ODI	0.22**	**−**0.43**	−0.68**	0.58**	0.69**	–
Mean PROMs	0.27**	0.64**	0.83**	0.77**	0.77**	0.85**
Overall MQO	0.86**	0.44**	0.55**	0.52**	0.62**	0.59**
Linear regression with constant (beta coefficients) for *t*1 scores (predictor FTF *t*1; *R*^2^ = 0.098;* p* = 0.000***; c = 18.371): −0.073*EQ-VAS *t*1 + −0.029*EQ5D TTO *t*1 + 0.074*NPRS *t*1 + 0.252*HAQ *t*1 + −0.036*ODI *t*1						
(**b**) Spearman correlations (rho) for *t*2; *n* = 395						
*t*2	FTF-CROM	EQ-VAS	EQ5D TTO	NPRS	HAQ	ODI
FTF-CROM	–					
EQ5D health (EQ-VAS)	−0.17**	–				
EQ5D TTO	−0.29**	0.45**	–			
NPRS	0.21**	−0.42**	−0.67**	–		
HAQ	0.36**	−0.41**	−0.73**	0.55**	–	
ODI	0.27**	−0.46**	−0.77**	0.64**	0.73**	–
Mean PROMs	0.32**	0.73**	0.84**	0.80**	0.80**	0.85**
Overall MQO	0.84**	0.53**	0.65**	0.57**	0.68**	0.65**
Linear regression with constant (beta coefficients) for *t*2 scores (predictor FTF *t*2; *R*^2^ = 0.139; *p* = 0.000***; *c* = 16.653): −0.029*EQ-VAS *t*2 + −0.082*EQ5D TTO *t*2 + 0.022*NPRS *t*2 + 0.254*HAQ *t*2 + 0.037*ODI *t*2						
(**c**) Spearman correlations (rho) for differences (*t*2 − *t*1; Δ); *n* = 395						
Difference; Δ (D = *t*2 − *t*1)	FTF-CROM	EQ-VAS	EQ5D TTO	NPRS	HAQ	ODI
FTF-CROM	–					
EQ5D health (EQ-VAS)	0.01	–				
EQ5D TTO	0.02	0.11*	–			
NPRS	0.05	−0.12*	−0.38**	–		
HAQ	−0.00	−0.08	−0.34**	0.24**	–	
ODI	0.02	−0.17**	−0.40**	0.37**	0.34**	–
Mean PROMs	**0.05**	0.54**	0.67**	0.66**	0.49**	0.62**
Overall MQO	0.60**	0.42**	0.49**	0.55**	0.36**	0.49**
Linear regression with constant (beta coefficients) for difference scores (predictor FTF difference; *R*^2^ = 0.003; *p* = 0.964; c = −4.528): −0.006*EQ-VAS D + 0.026 EQ5D TTO D + 0.023*NPRS D + 0.006*HAQ D + 0.037*ODI D						
(**d**) Spearman correlations (rho) for performance score (*t*2 + Δ; *t*2D); *n* = 395						
Performance scores	FTF-CROM	EQ-VAS	EQ5D TTO	NPRS	HAQ	ODI
FTF-CROM	–					
EQ5D health (EQ-VAS)	−0.11*	–				
EQ5D TTO	−0.15**	0.22**	–			
NPRS	0.13**	−0.26**	−0.48**	–		
HAQ	0.28**	−0.26**	−0.36**	0.40**		
ODI	0.23**	−0.33**	−0.50**	0.53**	0.24**	–
Mean PROMs	0.23**	0.68**	0.64**	0.73**	0.63**	0.75**
Overall MQO	0.74**	0.52**	0.51**	0.56**	0.56**	0.63**
Linear regression with constant (beta co + B2:H48ficients) for perf. scores (predictor FTF *t*2D; R^2^ = 0.093; *p* = 0.000***; c = 7.709): 0.001*EQ-VAS *t*2D + −0.028*EQ5D TTO *t*2D + −0.020*NPRS *t*2D + 0.191*HAQ *t*2D + 0.140*ODI *t*2D						

CROM-FTF, mean of PROMs and the overall medical outcome (MQO; mean of PROMs and CROM-FTF) are highlighted in bold

Although the level of significant correlations (*, ** or bold) was reached for multiple measures (all *p* < 0.05*)—with the exception of the difference values—the Spearman’s rho of the FTF with the PROMs was very weak to weak (rho = 0.00–0.36). A rho = 0.10 corresponds to a small effect, rho of 0.30 correspond to a middle effect, and rho values > 0.50, to a large effect size [36]

**Fig. 1:**
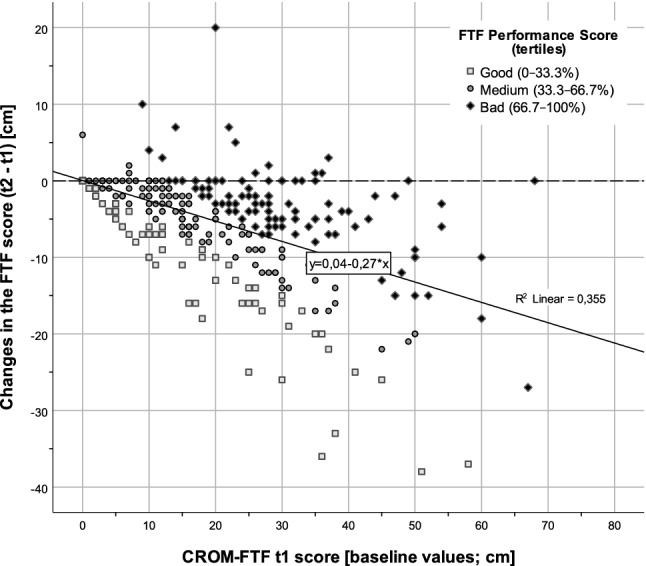
Baseline values (*t*1) and changes of CROM-FTF (*t*2 – *t*1). Changes of a Fingertip-To-Floor (FTF) test in relation to baseline values (*t*1) and the categorized performance score (*t*2 + Δ; tertiles highlighted in from and color). Tertiles were chosen because the expected and observed improvements in outcome measures (see Table 2) in inpatient rehabilitation are clearly visible in around 2/3 of patients [16]. Among the LBP patients, 73 had optimal values (0) for *t*1 and *t*2

**Fig. 2:**
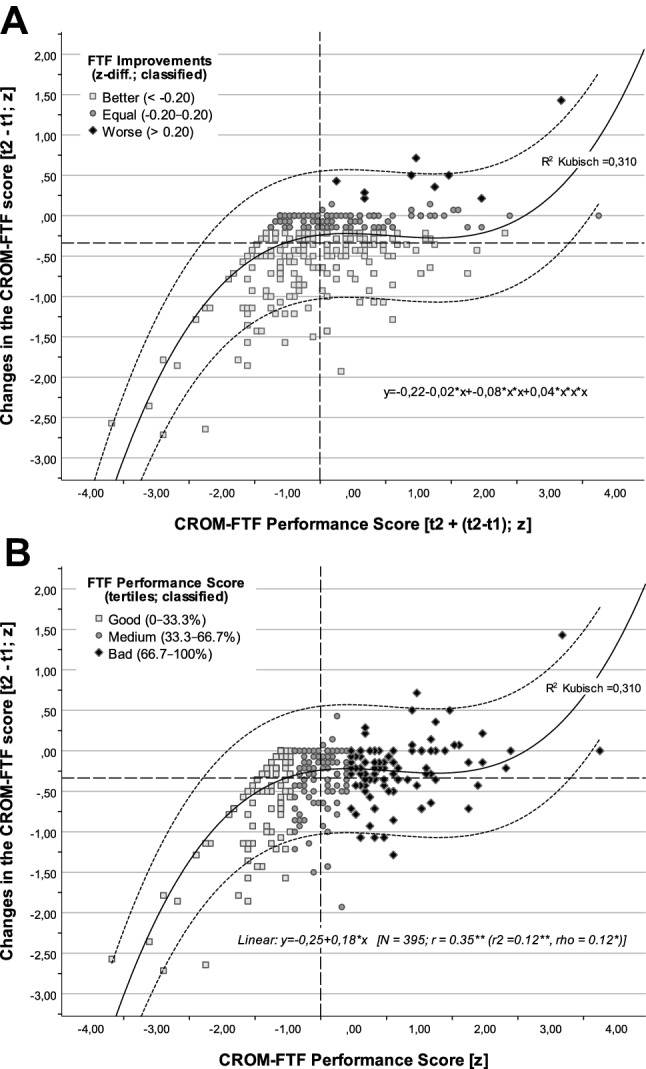
**ab** FTF improvements vs. performance score and changes of FTF. Classification for improvements is based on the value distributions (*z*-differences; SMD; **a** top). Difference values with no significant changes normally range randomly from 0.00 ± 0.20 (1/5 SD) [36]. Tertiles for FTF performance score were chosen, because the expected and observed improvements are around 2/3 (**b** bottom) [16]

**Fig. 3:**
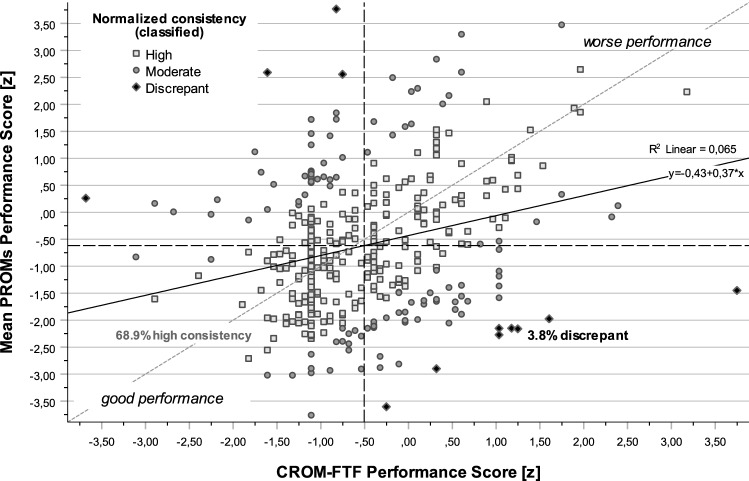
Consistency within CROM vs. PROMs performance scores. In most cases (68.9%), the performance scores between CROM-FTF and PROMs point in the same direction. Specifically, 27.3% of results in mean PROM performance scores showed moderate agreement in terms of performance with CROM-FTF. In 3.8% of the cases, the results between CROM-FTF vs PROMs were contrary to each other (discrepant)

Figure 1 shows changes in the results of a Fingertip-To-Floor (FTF) test as compared to baseline values (*t*1) and the categorized performance score. Based on this example of an objective measurement of physical mobility, a patient's performance could still be classified as good or medium, even if only a small or no increase could be measured during rehabilitation. Classification on improvements in Fig. 2 are based on the value distributions (changes; SMD; Fig. 2a; [16]) and tertiles for FTF performance score (Fig. 2b right). A cubic relationship (*r*^*2*^ = 0.31, *p* < 0.001) could be observed between the different perspectives of an outcome assessment. Based on the method of performance evaluation (*t*2D) a distinction was made between the high, moderate, or discrepant consistency of CROM-FTF and PROM outcomes (Table 4 and Fig. 3).

**Table 4:** Consistency of performance scores between outcome measurements

Consistency (*z-*differences; classified within/between tertiles)	CROM-FTF performance score (*t*2 + Δ) normalized with SD [*z*]			Performance score ± SD [*t*2z + Δz, *t*2D]			Diff. t2D (CROM − PROM)		
	High consistency (%)	Moderate consistency (%)	Discrepant (%)				Mean		SD
CROM-FTF *t*2 + Δ	100.0	0.0	0.0	**−**0.50	±	0.93	0.00	±	0.00
*Changes FTF (*t*2 –* t*1)*	*61.3*	*36.5*	*2.3*	*NaN*	±	*NaN*	*NaN*	±	*NaN*
*Random variable* t*2* + *Δ (0* ± *√5) no consistency*	*33.3*	*33.3*	*33.3*	*0.00*	±	*2.24*	*0.00*	±	*3.16*
EQ5D health (EQ-VAS) *t*2 + Δ	59.2	27.3	13.4	**−**0.40	±	1.93	**−**0.11	±	2.10
EQ5D TTO *t*2 + Δ	72.9	22.3	4.8	**−**0.50	±	1.45	0.00	±	1.55
NPRS *t*2 + Δ	57.5	34.7	7.8	**−**0.78	±	1.56	0.27	±	1.70
HAQ *t*2 + Δ	76.7	18.2	5.1	**−**0.22	±	1.34	**−**0.29	±	1.39
ODI *t*2 + Δ	71.4	24.8	3.8	**−**0.51	±	1.32	0.00	±	1.40
**Mean PROMs *t*2 + Δ**	**68.9**	**27.3**	**3.8**	**−0.62**	** ± **	**1.34**	0.11	±	1.42

CROM-FTF, mean of PROMs and the overall medical outcome (MQO; mean of PROMs and CROM-FTF) are highlighted in bold

Consistency. In most cases (68.9%), the results in CROM-FTF and PROMs point in the same direction (high consistency). Specifically, 27.3% of results in PROM scores showed a moderate agreement in performance with CROM-FTF (moderate consistency). In 3.8% of the cases, the results between CROM-FTF vs PROMs were contrary to each other (discrepant)

Consistency of performance (*t*2 + Δ; Differences of *t*2D/√10) using normal scores in three categories: (high consistency = *t*2D within one/same tertile. moderate consistency = between one and two tertiles. discrepant = more than two tertiles difference in normalized performance scores); n = 395

In most cases (68.9%), the performance scores between CROM-FTF and PROMs pointed in the same direction (Table 4). Specifically, 27.3% (18.2–34.7%) of results in PROM performance scores showed a moderate agreement in terms of performance with CROM-FTF. In 3.8% of the cases, the results between CROM vs PROMs were contrary to each other (discrepant). The strongest descriptive performance score was observed for NPRS (*t*2_z_ + Δ_z_ = −0.78 ± 1,56).

The stochastic test distribution of a performance score shows that a dependence exists between the *t*2_z_ + Δ_z_ with a *t*2_z_ of *r* = 0.90 and with a *t*1 of *r* = −0.45, respectively. This is quite similar to the dependence measured between *t*1 or *t*2 scores and the difference score (Δ_*;*_* r* = −0.70). A correlation of independent, normally distributed random variables between *t*2_z_ + Δ_z_ and changes (*t*2 − *t*1) would result in a correlation coefficient of 0.95.

For each outcome measure, score differences (Δ, changes) between the beginning (*t*1, pre-score) and the end (*t*2, post-score) of rehabilitation were calculated. These differences were tested to detect significant interactions with moderating factors like sex, age, the BMI, or ICD diagnoses (Table 5). The test results show that only BMI (η_p_^2^ = 0.041, *p* < 0.001) and baseline values (η_p_^2^ = 0.182, *p* < 0.001) served as critical success (between) factors that contributed to significant changes in outcome measurements. Obese LBP patients with a BMI > 30 and patients with poorer FTF-initial values (*t*1) showed the greatest improvements in CROM-FTF. The latter finding contrasts with the CROM performance score, where most patients who already had good initial values were rated as good "performers" (η_p_^2^ = 0.222, *p* < 0.001). As shown in Table 5, the method of performance evaluation (*t*2D) was much more sensitive to the individual factors as compared to the difference scores, since this method also depended on the actual functional status of the patient and rehabilitative clinical practice. Patients with poor functional status in PROMs received more individualized therapies (η_p_^2^ = 0.043, *p* < 0.01; not valid for CROM-FTF: *p* > 0.05). Younger male LBP patients could be expected to perform better in HAQ (age: η_p_^2^ = 0.040, *p* < 0.001; sex: η_p_^2^ = 0.011 *p* < 0.05), whereas female patients showed better performance in the FTF (η_p_^2^ = 0.016, *p* < 0.05). In addition to already good initial values (all *p* < 0.001; overall MQO: η_p_^2^ = 0.290), the BMI had the greatest influence on the performance evaluation (η_p_^2^ = 0.027, *p* < 0.01), because overweight patients still showed the worst performance in PROMs after orthopedic inpatient rehabilitation.

**Table 5:** Effect sizes for outcome measurements (changes, *t*2D) and moderating factors

η_p_^2^		Unifactorial part. Eta^2^ for changes (interaction)^*^						Main effect		Unifactorial part. Eta^2^ for performance scores^**^						
	*Between-factors*	Sex	Age	ICD	BMI	IT	Pre _MQO_	Time		Sex	Age	ICD	BMI	IT	Pre _MQO_	
*Outcome measures*		f/m	3-stage	6-stage	3-stage	3-stage	3-stage	2-stage		f/m	3-stage	6-stage	3-stage	3-stage	3-stage	
CROM (1)	FTF	0.000	0.001	0.010	0.041^***^	0.008	0.182^***^	0.328^***^		0.016^*^	0.009	0.008	0.033^**^	0.004	0.222^***^	
PROMs (6)	EQ5D Health (EQ-VAS)	0.000	0.006	0.009	0.002	0.001	0.007	0.059^***^		0.001	0.017^*^	0.015	0.001	0.005	0.044^***^	
	EQ5D TTO	0.003	0.008	0.001	0.009	0.007	0.010	0.133^***^		0.001	0.004	0.003	0.024^**^	0.035^**^	0.066^***^	
	NPRS	0.001	0.003	0.012	0.006	0.003	0.019^*^	0.251^***^		0.002	0.004	0.012	0.013	0.020^*^	0.045^***^	
	HAQ	0.000	0.003	0.007	0.015	0.008	0.005	0.058^***^		0.011^*^	0.040^***^	0.008	0.018^*^	0.030^**^	0.187^***^	
	ODI	0.011^*^	0.003	0.001	0.004	0.001	0.006	0.230^***^		0.002	0.006	0.004	0.012	0.030^**^	0.140^***^	
	Mean PROMs	0.003	0.002	0.004	0.009	0.005	0.008	0.302^***^		0.005	0.016^*^	0.009	0.021^*^	0.043^**^	0.150^***^	
overall MQO	PROMs and CROM-FTF	0.001	0.000	0.007	0.017^*^	0.001	0.124^***^	0.472^***^		0.001	0.017^*^	0.010	0.027^**^	0.024^*^	0.290^***^	
Consistency	Difference CROM − PROMs	0.002	0.003	0.006	0.029^**^	0.013	0.042^***^	0.009		0.022^**^	0.008	0.006	0.024^**^	0.034^**^	0.003^***^	

Sex (female, male); age (< = 50, 51–60, 61 +); ICD…six main-diagnoses (*n* > 30; M51.1, M51.2, M53.9, M54.4, M54.9, other LBG); BMI…Body Mass Index (< = 25, 25–30, 30 +); IT… Individual therapy minutes (< = 360, 361–450, 451 +); pre…pre-rehabilitation value of MQO (tertile)

A part. Eta^2^ (η_p_^2^) between 0.01 and 0.06 corresponds to a small effect, occurrences of 0.06–0.14 a middle effect and values > 0.14 a large effect; level of significance (bold): *p <  0.05, **p < 0.01, ***p < 0.001; *N* = 395 LBP patients

^*^Differences (improvements; post − pre) from admission to discharge (corresponds to the interaction: time x between-factor)

^**^Performance Scores: *t*2D = *t*2_z_ + (*t*2_z_ − *t*1_z_)

Performance scores were much more sensitive to the individual factors compared to the difference scores. The factors sex and age showed significant effects in performance scores. In addition to initial values (all *p* < 0.001; overall MQO: η_p_^2^ = 0.290), the BMI had the greatest influence on the performance evaluation (η_p_^2^ = 0.027, *p* < 0.01). The amount of individual therapy (IT) depends on the medical history, if the rehabilitation program is classified as follow-up treatment procedure after surgery (IT > 450 min)

## Discussion

In this retrospective cohort study, we analyzed the changes observed in outcome measures during LBP rehabilitation and introduced a new performance outcome measure. In terms of overall medical quality outcome, the rehabilitation resulted in a large effect size (Cohens *d* = 0.94). Medium effect sizes were observed for CROM-FTF (*d* = 0.70) and PROMs (*d* > 0.50). PROMs deteriorated in 13.9% of all LBP patients, while only 2.3% showed a deterioration in CROMs (Table 2). The correlations between PROMs and CROM-FTF were low to moderate, with the highest identified for HAQ-DI (rho = 0.30*–*0.36); regarding changes, no significant correlations could be shown (Table 3).

In previous studies, a number of factors were identified that influence the PROMs. The BMI has been reported to correlate with PROMs, including pain, in LBP patients, whereas no such correlation was found for the Timed Up-and-Go Test [37]. However, a systematic review of twin studies revealed that the association of LBP and obesity seems to be weak [38]. In LBP, females seem to report more pain, higher levels of disability and lower quality of life than males, even though the values of CROMs do not seem to differ [14]. Similar findings were reported in a recent study, suggesting that female gender, a lower education level and higher ODI scores predict worse outcomes after surgery for LBP [39]. Socioeconomic status and depression have also been identified as possible factors that predict a poor rehabilitation outcome [40].

Concerning the correlation between PROMs and CROMs, Melzer et al. [41] reported poor to moderate associations between performance-based measures and self-reported functional status in older patients. Obvious interdependencies exist between patient-reported outcomes and performance measures; however, these are used to evaluate different outcomes and to assess the effects of complementary therapeutic modalities in orthopedic rehabilitation. This finding is consistent with that of Stratford et al. [42], who proposed that self-reported outcomes and performance measures can be used to evaluate different aspects of physical functioning. The authors concluded that self-report measures provide information about the experience associated with the execution of the task, while performance measures contain information about the ability to complete the task [42]. It is, therefore, necessary to carry out performance-based tests to fully characterize the changes in the patients’ physical functions [43]. Performance-based tests like the FTF provide objective information about how the patients actually function, information that cannot be captured by PROMs alone. These CROMs of physical function allow healthcare staff to evaluate what individuals can actually do rather than what they perceive they can do; the latter is then assessed using PROMs.

Outcome measures and endpoints are often systematically associated with influences other than treatment and can be interpreted as both causes and responses, which may elicit different responses in different individuals. This leads to large variability and confounds the observed outcomes. Therefore, the level of agreement between PROMs and CROMs cannot be taken for granted. In addition to human factors and differences in baseline conditions between patients, methodological and conceptional issues such as ceiling effects, nonlinearity and reliability (cf. state vs. trait) play moderating roles in observable associations between PROMs and CROMs. Outcome measures are influenced by daily activities, underlying conditions and personal factors such as educational level, mood [13], patient hospital experience, overall satisfaction, personal expectations [44] and gender [14, 45]. Temporal aspects should also not be neglected, such as retest reliability and the characteristics of the methods and outcome variables used. It is reasonable to assume that an aggregate outcome, such as a generic measure used to quantify patient disability, may have more stable characteristics or be subject to less variation than some other disease-specific measures, such as self-reports of pain conditions. This is true for both CROMs and PROMs. In this study, the FTF (*r *_*t*1 to *t*2_ = 0.90) showed high reliability, as did the ODI (*r* = 0.80) and HAQ-DI (*r* = 0.83), whereas the EQ-VAS (*r* = 0.43) and NPRS (*r* = 0.57) showed lower stability over time. In addition to the associations between outcomes, this obviously has implications for the responsiveness of the measures. Each approach has its strengths in this regard and should be considered when designing clinical trials. Lower test–retest correlations can also be observed for "objective" physiological measures such as heart rate or diastolic blood pressure [46]. Hamilton et al. [47, 48] indicated that lower confidence in ‘subjective’ PROMs as compared to ‘objective’ clinical measures is not justified, stating *“… we would expect to see a similar direction of change in the respective scores when measuring the effect of an intervention, but to expect the same result misunderstands that PROMs capture a different aspect of outcome than a performance test does. The relationship between assessment of performance and report of performance improved as the patient's report of pain diminished, suggesting that patients' reporting of functional outcome after TKR is influenced more by their pain level than their ability to accomplish tasks.”*

Unlike the commonly used methods, the performance scores in the PROMs showed significant correlations with the FTF performance scores (Table 3). This method of performance evaluation (*t*2D) was much more sensitive to the individual factors as compared to the difference scores (Table 5), since the medical evaluation also depends on the patient’s actual functional status and the rehabilitative clinical practice (Fig. 1). In a theoretical sample with independent, normally distributed random variables, a regression analysis of *t*2_z_ + Δ_z_ and *t*2 − *t*1 would result in a maximum correlation coefficient of 0.95. In the present sample, this coefficient could not be achieved (e.g., for mean PROMs: rho = 0.70), possibly due to the interdependency of outcome measures within a subject, smaller variances and the desired effects of the intervention.

In a minority (13.9%) of patients, the PROMs worsened significantly over time. Although this finding cannot be readily explained, it may have been caused in part by ceiling effects, as these patients usually displayed relatively good outcome values at the beginning of rehabilitation (Table 5). In the future, research should be carried out to identify the cause of these differences by more thoroughly comparing the consistent and discrepant results within a patient.

In more than half of the cases, high consistency levels were observed among the newly introduced performance scores between CROM-FTF and PROMs (Table 4). Further studies would be needed to identify critical success factors and non-responders in the rehabilitation process. Simply looking at the changes in outcome measures does not seem to allow healthcare professionals to detect such factors. The calculation of the performance score presented in this paper provides a promising alternative approach, as it takes into account a classification of the patient’s health status after rehabilitation (*t*2), on the one hand, and the patient’s progress made during the rehabilitation process (changes; Δ), on the other.

## Limitations

For ethical, practical and economic reasons, it was not possible to include a randomized control group in this study. Due to the study design, causal conclusions must be drawn with caution. The beneficial effects resulting from participation in an inpatient rehabilitation may not be sustainable once the patients return to their usual everyday lives [49]. However, even minor changes in lifestyle can lead to functional adaptations and the normalization of physiological functions, which help the patient to recover from chronic inflammatory or degenerative diseases [50].

## Conclusions

Rehabilitation plays a vital role in preventing and minimizing the functional limitations associated with ageing and chronic conditions. Strong evidence supports the argument that inpatient rehabilitation is a necessary part of the treatment of inflammatory and degenerative diseases, as well as functional limitations after surgery. Despite the large international differences observed in terms of the variety of the composition of teams involved in rehabilitation and the implemented treatment measures, the observed strong effect sizes can support individual evaluation. These effect sizes allow medical professionals and researchers to compare health programs and developments in prevention, healthcare and rehabilitation. More importantly, they allow them to more effectively improve the wellbeing of patients with chronic conditions. Due to the use of standardized service portfolios, external reviews and the fact that insurers centrally control the assignment of modalities, we assume that the initial values and outcomes are representative for an orthopedic inpatient rehabilitation program in Austria.

An improvement or decline in the CROM did not serve as an indicator for what patients reported about their perceived functioning or pain. A deterioration in self-reported outcomes in 13.9% of all LBP patients during rehabilitation was observed. These changes alone do not reflect clinical evaluation practice, because they may not detect non-responders and the respective critical factors. They are usually not significantly influenced by known moderating (critical individual) factors. The new method presented in this work to assess individual “performance scores” within a patient can be used effectively to identify critical success factors and non-responders in the rehabilitation process, in a simple and user-friendly way. Further research is warranted, in order to ascertain the usefulness of this new method in other patient populations and treatments, as well as its utility in predicting long-term success and optimizing current rehabilitative practice.

## Data Availability

The datasets used and/or analyzed during the current study are available from the corresponding author upon reasonable request.

## Abbreviations

CI: Confidence interval
CROM: Clinician-reported outcome measure
*d*: Cohen’s *d* (effect size)
EQ5D: European Quality of Life-5 Dimensions (questionnaire)
EQ-VAS: VAS valuations of EQ-5D (self-rated health in %; 0–100)
HAQ-DI: Health Assessment Questionnaire Disability Index
MQO: Medical quality outcome
*N* (*n*): Sample size
NPRS: Numerical pain rating scale
*p*: Significance level (risk of error)
PROM: Patient-reported outcome measure
*r*: Correlation coefficient (Pearson)
rho: Spearman’s rank correlation
SD: Standard deviation
SMD: Standardized mean difference
*t*1: Pre-test (baseline) score
*t*2: Post-test (end of rehabilitation) score
*t*2D: Performance score (*t*2 + Δ)
TTO: Time trade-off (EQ5D valuation technique)
VAS: Visual analogue scale
*z*: *z*-Value, standard score (scale: 0 ± 1; mean ± SD)
η_p_^2^: Partial eta^2^ (effect size)
